# Comparative analysis of *Thalassionema* chloroplast genomes revealed hidden biodiversity

**DOI:** 10.1186/s12864-022-08532-6

**Published:** 2022-04-27

**Authors:** Mengjia Zhang, Nansheng Chen

**Affiliations:** 1 CAS Key Laboratory of Marine Ecology and Environmental Sciences, Institute of Oceanology, Chinese Academy of Sciences, Qingdao, 266071 China; 2 Laboratory of Marine Ecology and Environmental Science, Qingdao National Laboratory for Marine Science and Technology, Qingdao, 266200 China; 3 College of Marine Science, University of Chinese Academy of Sciences, Beijing, 10039 China; 4 Center for Ocean Mega-Science, Chinese Academy of Sciences, Qingdao, 266071 China; 5 Department of Molecular Biology and Biochemistry, Simon Fraser University, 8888 University Drive, Burnaby, BC V5A 1S6 Canada

**Keywords:** *Thalassionema* species, Chloroplast genome, Comparative genomics, Divergence time

## Abstract

The cosmopolitan *Thalassionema* species are often dominant components of the plankton diatom flora and sediment diatom assemblages in all but the Polar regions, making important ecological contribution to primary productivity. Historical studies concentrated on their indicative function for the marine environment based primarily on morphological features and essentially ignored their genomic information, hindering in-depth investigation on *Thalassionema* biodiversity. In this project, we constructed the complete chloroplast genomes (cpDNAs) of seven *Thalassionema* strains representing three different species, which were also the first cpDNAs constructed for any species in the order Thalassionematales that includes 35 reported species and varieties. The sizes of these *Thalassionema* cpDNAs, which showed typical quadripartite structures, varied from 124,127 bp to 140,121 bp. Comparative analysis revealed that *Thalassionema* cpDNAs possess conserved gene content inter-species and intra-species, along with several gene losses and transfers. Besides, their cpDNAs also have expanded inverted repeat regions (IRs) and preserve large intergenic spacers compared to other diatom cpDNAs. In addition, substantial genome rearrangements were discovered not only among different *Thalassionema* species but also among strains of a same species *T. frauenfeldii*, suggesting much higher diversity than previous reports. In addition to confirming the phylogenetic position of *Thalassionema* species, this study also estimated their emergence time at approximately 38 Mya. The availability of the *Thalassionema* species cpDNAs not only helps understand the *Thalassionema* species, but also facilitates phylogenetic analysis of diatoms.

**Supplementary Information:**

The online version contains supplementary material available at 10.1186/s12864-022-08532-6.

## Introduction

The diatom genus *Thalassionema* (Grunow) Mereschkowsky belongs to family Thalassionemataceae, order Thalassionematales, class Bacillariophyceae, and phylum Bacillariophyta [1]. It contains more than 19 taxa, three of which are frequently observed in the China coastal regions, including *T. nitzschioides*, *T. bacillare*, and *T. frauenfeldii* [1, 2]. This genus is taxonomically defined by its rectangular cells, which are straight in girdle view, with small and numerous plastids. The cells have one marginal row of areolae on the valve face or mantle junction of each valve, and have one rimoportula at each of the valve ends with external opening located on the apical mantle or valve face [2–5]. To identify *Thalassionema* at the species level, many morphological characteristics, such as valve apices, length, width, marginal areolae density, areolar occlusions, marginal foramina shape and rimoportula placement are often measured [5–7]. The *Thalassionema* species are cosmopolitan in all but the Polar regions, they often occur in large abundance and are dominant components of the plankton diatom flora [7–9].

As is known that diatoms carry out about one-fifth of the total photosynthesis on the earth, the widespread *Thalassionema* species are not exceptions, providing considerable primary productivity [10]. The large quantity, on the other hand, has led some *Thalassionema* species to form harmful algal blooms (HABs) in China, like *T. nitzschioides* var. *nitzschioides* bloom in Dapeng Bay in 1992 [11, 12]. In addition, *Thalassionema* species are heavily silicified, thus are abundant in pelagic and hemipelagic sediments and are dominant constituents of sediment diatom assemblages [7]. Because of the wide distribution, the abundance in sediments, and the long stratigraphic ranges, *Thalassionema* genus is an ideal indicator for studying the modern gyral circulation systems, the surface water masses, and the paleo-temperature [7, 13, 14]. As a result, most researches about *Thalassionema* species so far have focused on their indicative function based on morphological features, while little is known about the species themselves, especially about their phylogenetic relationship [7, 13, 14]. Their molecular information is now limited to only several common molecular markers [15, 16].

For phylogenomic research, chloroplast genome (cpDNA) is an ideal super-barcode, in that it is mostly composed of single copy genes with few horizontal transfer events [17]. Besides, for a wide range of diatoms, plastid protein-coding genes (PCGs) are easily aligned [18]. To date, cpDNA has been widely used as a source of valuable data for understanding evolutionary biology on plants, and are increasingly applied to species classification and identification, as well as studying the complex evolutionary relationships of algal species [19–23].

In this project, we constructed the cpDNAs of seven *Thalassionema* strains collected from South China Sea, which represented three common species in Chinese coastal regions. They are also the first cpDNAs for the entire order Thalassionematales. We carried out inter-species and intra-species comparisons of cpDNAs, uncovering interesting gene loss and transfer events, expansion and contraction of inverted repeat regions (IRs) and intergenic spacers, as well as substantial genome rearrangement events. We also confirmed the phylogenetic positions of *Thalassionema* species and estimated their emergence time, gaining insight into the evolution of *Thalassionema* species.

## Materials and methods

### Strain isolation and culturing

**Fig. 1:**
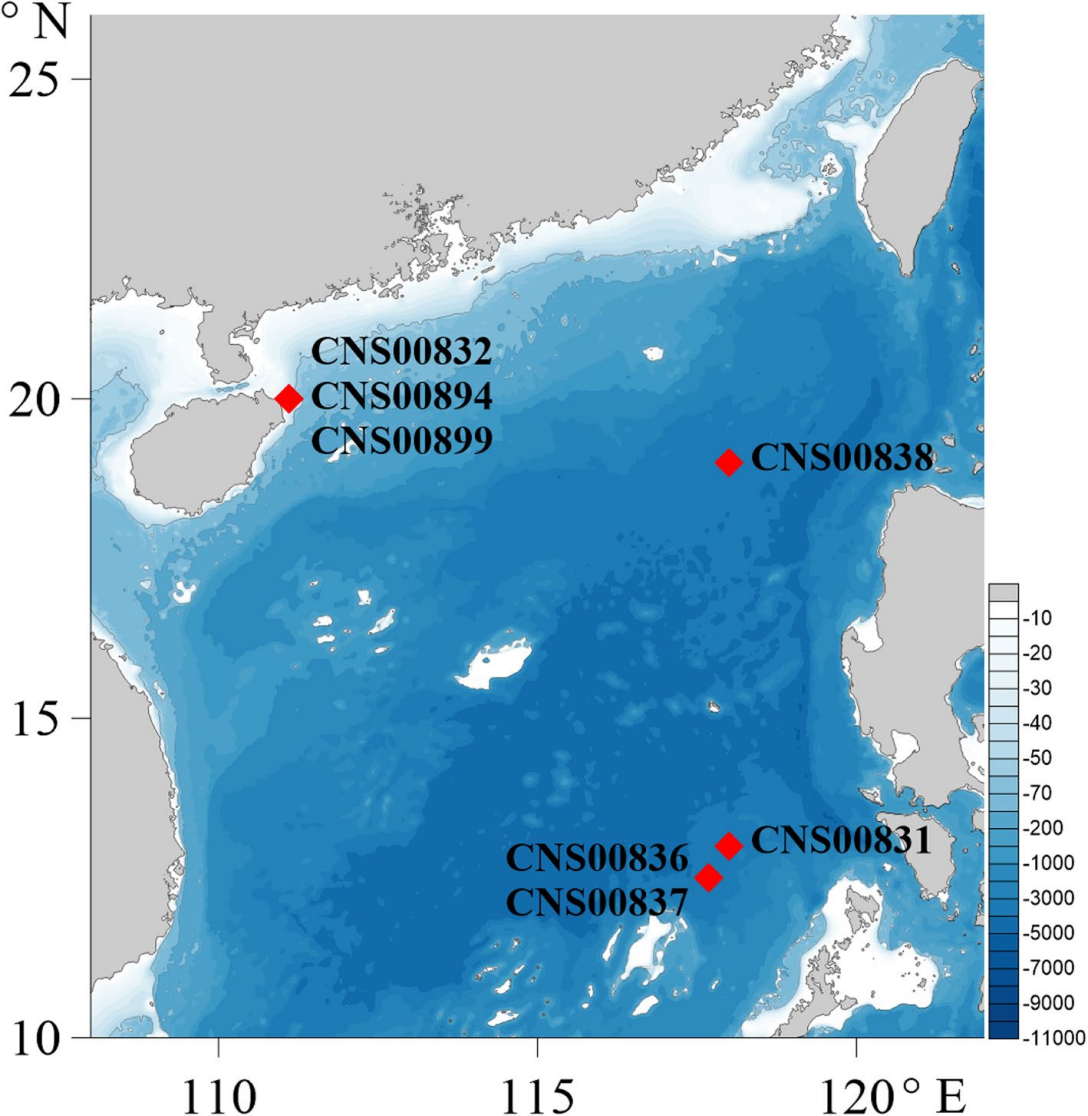
Sampling sites of seven *Thalassionema* strains analyzed in this study

Seven putative *Thalassionema* strains were isolated from seawater samples collected during an expedition in the South China Sea (May–June, 2021) on the research vehicle “TAN KAH KEE” supported by the Southern Marine Science and Engineering Guangdong Laboratory (Zhuhai) (Fig. 1). Briefly, phytoplankton cells were individually selected with a micropipette, followed by repeated washes before being transferred to 24-well culture dishes. They were then transferred to cell culture flask (60 ml to 750 ml) after about a week to accumulate enough biomass for further molecular assays. Phytoplankton cells were grown in L1 seawater culture medium [24] and maintained with temperature of 23–25 °C, irradiance of 30 μM photons m^−2^ s^−1^, and photoperiod of 12/12-h light/dark. Cultures at the exponential growth phase were harvested and concentrated via centrifugation, followed by total nucleic acids extraction with TIANGEN DNAsecure Plant Kit (TIANGEN, DP121221). The specimens were deposited in the collection of marine algae in KLMEES of IOCAS (Nansheng Chen, chenn@qdio.ac.cn) under the voucher number CNS00831, CNS00832, CNS00836, CNS00837, CNS00838, CNS00894, and CNS00899.

### DNA library preparation and genome sequencing

Each genomic DNA sample was fragmented by sonication via set program to a size of about 350 bp. Then a single adenosine "A" was added to the 3' end of the double-stranded DNA after end modification to prevent the self-connection of the flat ends between DNA fragments, and it can also highlight the complementary pairing with the single "T" at the 5' end of the next sequencing connector for accurate connection, effectively reducing the self-connection between library fragments. DNA fragments were then ligated with the full-length adapters for Illumina sequencing, followed by further PCR amplification. After PCR products were purified by AMPure XP system (Beckman Coulter, Beverly, USA), DNA concentration was measured by Qubit®3.0 Flurometer (Invitrogen, USA), libraries were analyzed for size distribution by NGS3K/Caliper and quantified by real-time PCR (3 nM). After cluster generation, the DNA libraries were sequenced on Illumina Novaseq 6000 platform and 150 bp paired-end reads were generated. Genome sequencing was finished at Novogene (Beijing, China). Raw sequencing data were filtered into clean data with FASTQ following the rules (1) identifying and removing reads with tail pollution; (2) removing reads with low quality (> 50% bases having Phred quality < 5) and (3) removing reads with ≥ 10% unidentified nucleotides (N). Due to the different genome sizes, the coverage depths were variable, ranging from 23 × to 98 × coverage of whole genomes (Table S1).

### Strain identification

Identification of the cultured *Thalassionema* strains was done according to both morphological observation and molecular identification. For morphological observation, cells were mounted on the glass-slide and observed with a ZEISS IMAGER A2 microscope equipped with differential interference contrast optics. For molecular identification, full-length 18S rDNA was assembled from the clean data using GetOrganelle (v1.7.5) [25] and SPAdes (v3.14.0) [26], with publicly available 18S rDNA of *Thalassionema* species serving as reference sequences. The assembled sequences were validated by the following steps. (1) Aligning reads to the assembled sequences using BWA (v0.7.17-r1188) [27]. (2) Extracting alignment results using SAMtools (v1.10) [28]. (3) Inspecting and correcting errors using IGV (v2.7.2) [29]. The evolutionary relationship of *Thalassionema* species based on full-length 18S rDNA was inferred using maximum likelihood (ML) method, conducted by MEGA (v7.0). The species *Synedra acus* (KF959659.1) was chosen as the outgroup taxa.

### Chloroplast genomes assembly and annotation

The complete cpDNAs were assembled from clean data using GetOrganelle (v1.7.5) [25] with the *Synedra acus* cpDNA (JQ088178) [30] serving as reference. The final version of each cpDNA was validated using the same method used for verifying full-length 18S rDNA described above in 2.3. The cpDNAs were first annotated using MFannot (https://github.com/BFL-lab/Mfannot) with genetic code of Bacterial, Archaeal and Plant chloroplast. Open Reading Frame Finder (ORF finder) (https://www.ncbi.nlm.nih.gov/orffinder) and BLAST similarity searches of the non-redundant databases at NCBI [31] were then applied to examine and edit gene models. Additionally, rRNA genes were identified using RNAmmer (v1.2) [32] and Barrnap (v0.9). The annotation results were further validated and formatted using NCBI’s Sequin (v16.0). The gene maps of the circular cpDNAs of *Thalassionema* species were generated with Organellar Genome DRAW (OGDraw) [33].

### Inter-species and intra-species genome comparison

The missing genes in cpDNAs of *Thalassionema* species were searched in genome assemblies based on Illumina reads using BLASTN (v2.12.0). The typical signal peptides were estimated using SignalP (v6.0). The expansions and contractions of IRs in cpDNAs were analyzed using irscope_pack.31 [34] and OGDraw. The intergenic spaces of cpDNAs were calculated and visualized using the R packages ggplot2 and reshape2 [35].

### Phylogenetic analysis of cpDNAs and estimation of divergence time

PCGs were extracted from the cpDNAs using BedTools (v2.28.0) [36]. PCGs shared by all 62 diatoms were then aligned using MAFFT (v7.471–1) [37] with default parameters. The ambiguously aligned regions in each alignment were removed using trimAl (v1.4) [38] with the option gt = 1, and all genes from each diatom were then concatenated with the same order using Phyutility (v2.7.1) [39]. The set of PCGs shared by the 62 Bacillariophyta cpDNAs were used for phylogenetic analysis, with *Triparma laevis* (AP014625) (Bolidophyceae, Ochrophyta) serving as the outgroup taxa [40]. The evolutionary relationship was inferred using ML method, conducted by IQ-TREE (v1.6.12) [41] with 1000 bootstrap replicates. The best-fit models for each partition were determined automatically using IQ-TREE with the subroutine ModelFinder. Multiple sequence alignments of complete cpDNAs were performed by Mauve Genome Alignment (v2.3.1) [42] with progressive Mauve algorithm. Pairwise comparisons were visualized using CIRCOS (v0.69) [43].

Divergence time estimation was performed by the set of PCGs shared in 28 Bacillariophyta cpDNAs using MCMCTree in PAML (v4.8a) [44]. Branch lengths, gradient (g) and Hessian (H) were estimated using maximum likelihood estimates (MLE) and GTR + G substitution model (model = 7) with independent rates clock model (clock = 2). Three calibration points (http://www.timetree.org/) were used in this analysis, including the calibration point between *Ectocarpus siliculosus* and diatoms (176.0–202.0 Million years ago (Mya)), the calibration point between *Rhizosolenia setigera* and *Skeletonema pseudocostatum* (90.5–91.5 Mya), and the calibration point between *Pseudo-nitzschia multiseries* and *Fragilariopsis cylindrus* (10.0–35.3 Mya). Tree files were visualized with Figtree (v1.4.3).

## Results

### Morphological and molecular identification of seven *Thalassionema* strains

**Fig. 2:**
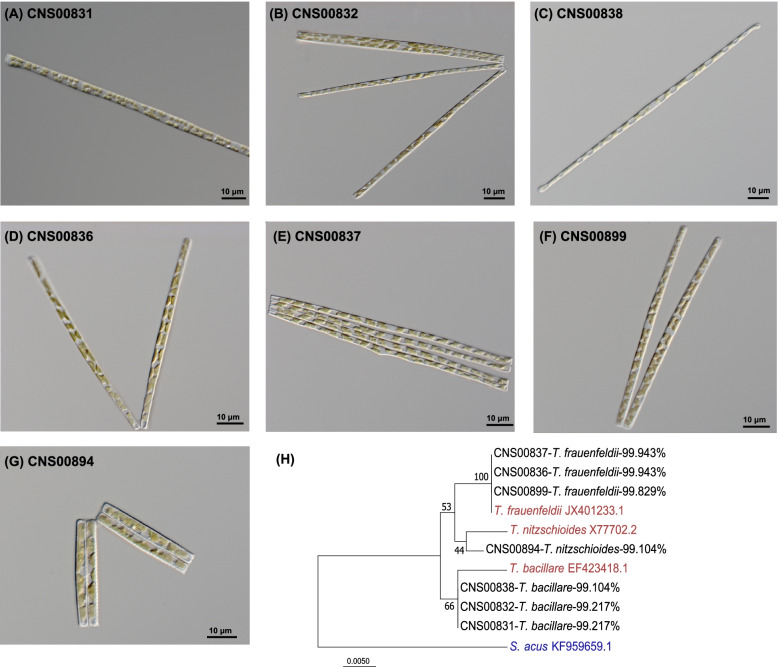
Morphological and molecular identification of seven *Thalassionema* strains. (**A-G)** Micrographs of seven *Thalassionema* strains (broad girdle view, live material DIC). (**H)** Phylogenetic tree based on maximum likelihood (ML) analysis of 18S rDNA gene of *Thalassionema* strains. *Thalassionema* species were used as references (red) and *S. acus* was used as out-group taxa (blue)

The seven strains (CNS00831, CNS00832, CNS00836, CNS00837, CNS00838, CNS00894, CNS00899) studied in this project were chosen based on the similarity of their morphological features to that of *Thalassionema* species. They were all rodlike in the gridle view with small, numerous plastids. Adjacent cells can be joined by colloid to form serrated or stellate groups (Fig. 2A-G), consistent with previous observations of the genus *Thalassionema* [2]. Among them, strain CNS00894 was annotated as *T. nitzschioides* because it is apparently shorter and more blunt in both sides (Fig. 2G**)**, which are distinguishing features of *T. nitzschioides* [2]. The other six strains could not be annotated to specific species for subtle morphological variations (Fig. 2A-F**)**.

We further examined all the strains by comparing their common molecular marker sequences (full-length 18S rDNA) with reference sequences. The strain CNS00894 was further confirmed to be *T. nitzschioides*, and other six strains were identified to two *Thalassionema* species, namely *T. bacillare* (CNS00831, CNS00832, and CNS00838) and *T. frauenfeldii* (CNS00836, CNS00837, and CNS00899). Phylogenetic analysis of 18S rDNA sequences indicated that all strains clustered well with corresponding *Thalassionema* reference sequences downloaded from GenBank (Fig. 2H), further confirming that these strains were indeed *Thalassionema* species.

### General characteristics of *Thalassionema* cpDNAs

**Fig. 3:**
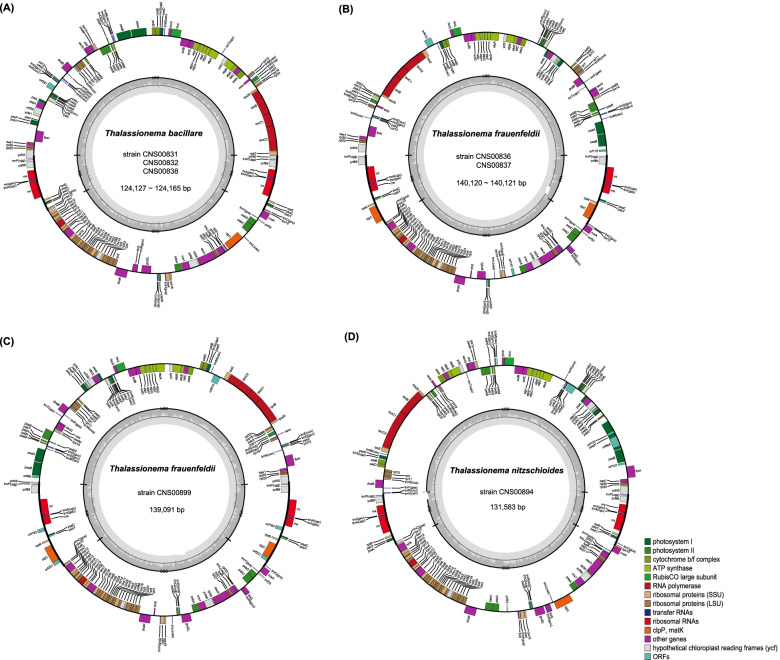
Gene maps of cpDNAs of seven *Thalassionema* strains. Genes shown on the inside of the map are transcribed in a clockwise direction, whereas those on the outside of the map are transcribed counterclockwise. The assignment of genes into different functional groups is indicated by different colors. The ring of bar graphs on the inner circle shows the GC content in dark gray

We constructed full-length cpDNAs of these seven *Thalassionema* strains for the first time, and these cpDNAs represented the first instances of cpDNAs of any *Thalassionema* species. They were all circular modules with varying lengths, ranging from 124,127 bp to 140,121 bp (Fig. 3). The cpDNAs of *T. frauenfeldii* were relatively longer than these of *T. nitzschioides* strains, and they were both longer than the *T. bacillare* cpDNAs (Table 1)*.* The GC contents of all seven strains were quite similar (29.01%-29.84). These *Thalassionema* cpDNAs all formed typical quadripartite structure with two inverted repeats regions (IRa, IRb), a large single copy (LSC) region, and a small single copy (SSC) region (Fig. 3). The proportion of each region in the cpDNA showed substantial variations among different *Thalassionema* species. Briefly, the *T. frauenfeldii* strains possessed the longest cpDNAs (139,091–140,121 bp), and had the longest IR and LSC regions. In contrast, *T. bacillare* possessed the shortest LSC and SSC regions of species, which contributed to their shortest cpDNAs. Notably, strain CNS00899, which was also annotated as *T. frauenfeldii* based on 18S rDNA, did not follow the above structural features for other *T. frauenfeldii* cpDNAs, suggesting potential genomic difference among these *T. frauenfeldii* strains.

**Table 1:** Chloroplast Genome Features of *Thalassionema*

Species	*T. bacillare*			*T. frauenfeldii*			*T. nitzschioides*	*S. acus*
Srtains	CNS00831	CNS00832	CNS00838	CNS00836	CNS00837	CNS00899	CNS00894	-
GenBank ID	OK574455	OK637332	OK637334	OK574456	OK637333	OK637335	OK574457	JQ088178
**Size /bp**								
Total (%GC)	124,165 (29.78%)	124,127 (29.79%)	124,131 (29.78%)	140,121 (29.84%)	140,120 (29.84%)	139,091 (29.68%)	131,583 (29.01%)	116,251 (30.57%)
IRA	9470	9451	9453	13,282	13,282	15,881	9052	6796
IRB	9470	9451	9453	13,282	13,282	15,880	9050	6795
LSC	62,496	62,496	62,496	69,833	69,832	66,603	67,639	61,723
SSC	42,729	42,729	42,729	43,724	43,724	40,727	45,842	40,937
**Gene content**								
Total numbers of genes	151	151	151	154	156	155	155	160
PCGs	120	120	120	121	121	121	121	127
Total number of introns	0	0	0	0	0	0	1 (in *psaA*)	0
ORFs	*orf455*	*orf455*	*orf455*	*orf410*, *orf116*, *orf157*	*orf410*, *orf116*, *orf157*, *orf342*, *orf119*	*orf410*, *orf99*, *orf193* and *orf201* (in IRs)	*orf452*, *orf107*, orf608 and orf123 (in intron)	*orf436*
tRNA genes	27	27	27	27	27	27	27	27
rRNA genes	*rnl*, *rns*	*rnl*, *rns*	*rnl*, *rns*	*rnl*, *rns*	*rnl*, *rns*	*rnl*, *rns*	*rnl*, *rns*	*rnl*, *rns*, *rrn5*
Other RNAs	*ssra*	*ssra*	*ssra*	*ssra*	*ssra*	*ssra*	*ssra*	*ssra*, *ffs*
Coding sequence	79.16%	79.68%	79.67%	73.99%	74.77%	75.62%	78.27%	88.63%
numbers of genes in IRS	12	12	12	12	11	14	11	8
**Intergenic spacer /bp**								
Maximum	986	986	986	2037	2037	1580	2174	324
Minimum	2	2	2	2	2	2	2	0
Average	163.06	157.94	157.96	224.62	215.24	206.39	179.27	78.91

Genes duplicated in the IR are only counted once

**Fig. 4:**
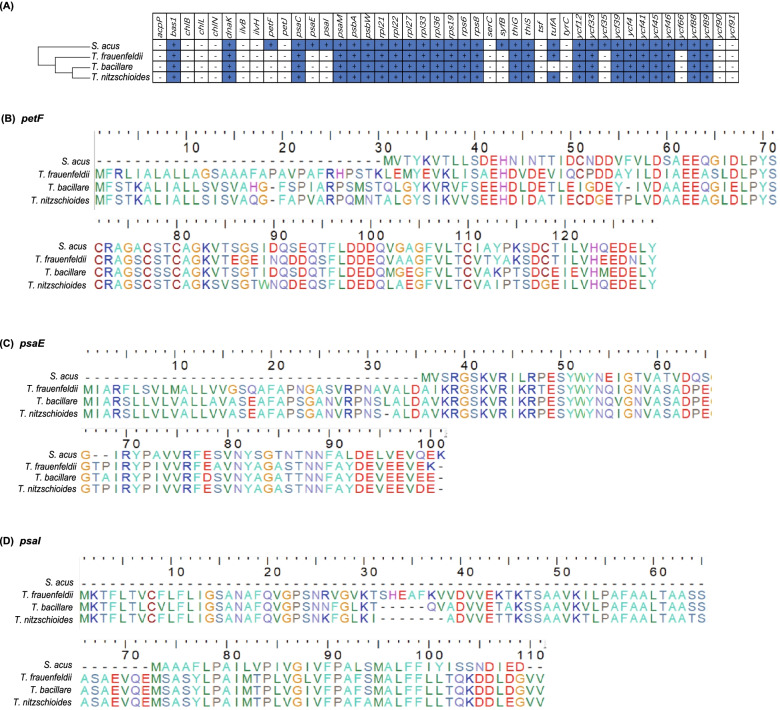
Gene losses and transfers of the cpDNAs of three *Thalassionema* species compared to *S. acus* cpDNA. (**A)** Presence and absence of 44 PCGs that used to be found lost in diatom cpDNAs in *Thalassionema* cpDNAs. Blue squares represent the presence of the gene, and white squares indicate the absence of the gene. (**B-D)** Protein sequences alignments of gene *petF*, *psaE* and *psaI* in the cpDNA of *S. acus* and in the nuclear genomes from three *Thalassionema* species, respectively

Although the sizes of cpDNAs of three *Thalassionema* species varied substantially, they had highly similar gene contents with only three differences. First, while the gene *tufA* was found in cpDNAs of *T. frauenfeldii* and *T. nitzschioides* strains, it was missing from the cpDNA of *T. bacillare* (Fig. 3, Fig. 4A). Second, a group II intron was found in the gene *psaA* in *T. nitzschioides* cpDNA (Table 1). Interestingly, a group II intron was also found in the same gene in cpDNA of the diatom *Toxarium undulatum* [45]. The intron was 2931 bp in size and encoded two open reading frames (*orf*s) (*orf608* and *orf123*). In contrast, no introns were found in cpDNAs of other *Thalassionema* strains. Third, a number of non-intron *orf*s were found in the cpDNAs of these *Thalassionema* strains, including both conserved *orf*s and strain-specific *orf*s. An orthologous *orf* was found to be conserved in the cpDNAs of all seven *Thalassionema* strains with slightly different lengths, which was *orf455* in *T. bacillare* strains (CNS00831, CNS00832, and CNS00838), *orf410* in *T. frauenfeldii* strains (CNS00836, CNS00837, and CNS00899), and *orf452* in the *T. nitzschioides* strain (CNS00894). Another orthologous *orf* was found to be conserved in the cpDNAs of four *Thalassionema* strains, which was *orf116* in CNS00836 and CNS00837 and *orf99* in CNS00899 of *T. frauenfeldii,* and *orf107* in CNS00894 of *T. nitzschioides*, and absent from *T. bacillare*. Among three strains of *T. frauenfeldii*, two strains (CNS00836 and CNS00837) contained *orf157*, and one strain (CNS00899) obtained unique *orf193* and *orf201* in its IRs. Additionally, CNS00837 obtained *orf119* and *orf342* that were absent from other *Thalassionema* strains (Table 1). All seven *Thalassionema* cpDNAs contained 27 tRNA genes, four rRNA genes (*rnl* and *rns* in IRs) and one tmRNA (*ssra*) (Table 1). The cpDNAs sequences of seven *Thalassionema* strains (CNS00831, CNS00832, CNS00836, CNS00837, CNS00838, CNS00894, and CNS00899) have been deposited in GenBank under accession numbers OK574455, OK637332, OK574456, OK637333, OK637334, OK574457 and OK637335, respectively.

### Comparative analysis of the cpDNAs

Comparative analysis of cpDNAs among these seven strains of three *Thalassionema* species, together with that of *S. acus*, which is the closest known diatom species whose cpDNA has been constructed, revealed that *Thalassionema* species possessed longer cpDNAs and some regions (IR, LSC, and SSC), while the length of coding sequences were unexpectedly shorter (Table 1).

Six genes were found missing from the cpDNAs of *Thalassionema* species compared to *S. acus* cpDNA, including *petF*, *psaE*, *psaI*, *syfB*, *ycf35*, and *ycf66* (Fig. 4A). Among these genes, the gene *petF*, which encodes ferredoxin, has been found either to be in the cpDNA or being transferred to the nuclear genome in phytoplankton, and the nuclear *petF* was likely obtained via endosymbiotic gene transfer (EGT) in *Thalassiosira* species [46]. As *petF* was not found in the cpDNAs of *Thalassionema* strains, we searched for candidate *petF* genes in the assembled genome sequences, which resulted in the identification of putative *petF* genes whose encoded peptides showing high similarity to *petF*-encoded protein (62.8%-72.2%) (Fig. 4B). Furthermore, typical signal peptides were found at the N-terminus of each nuclear *petF*-encoded protein, suggesting that nuclear *petF* genes in *Thalassionema* were acquired via EGT, and that nuclear *petF*-encoded proteins were transported to plastids. Similar results were found for *psaE* and *psaI* (Fig. 4C-D). Nevertheless, *syfB*, *ycf35*, and *ycf66* were not found in their corresponding nuclear genome assemblies, suggesting that these two genes may have been lost in evolution.

**Fig. 5:**
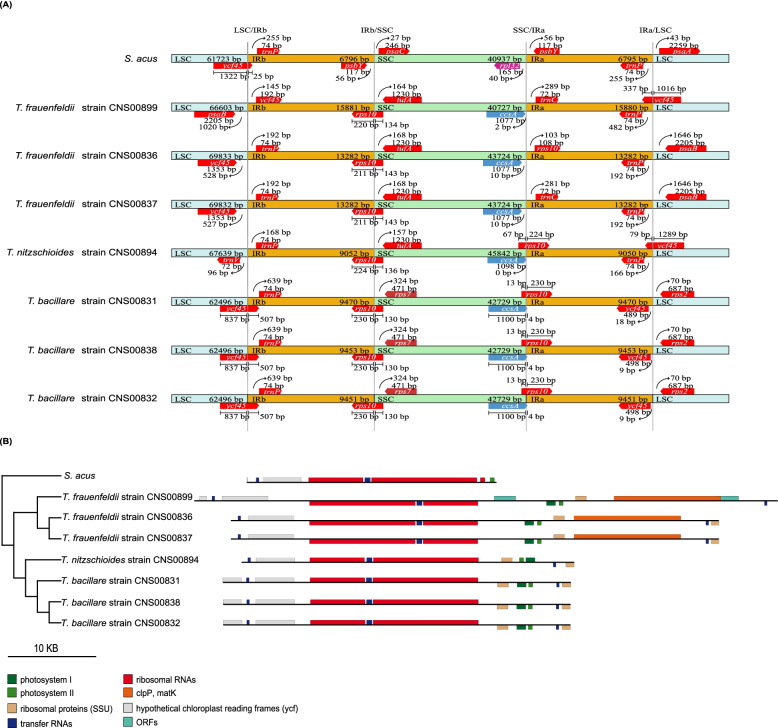
Expansion of IR regions in cpDNAs of seven *Thalassionema* strains and *S. acus.* **(A)** Comparative analysis of the boundaries of LSC, SSC and IR regions. (**B)** Comparative analysis of the length and components of IR regions

We analyzed the expansion of IR regions in cpDNAs of all seven *Thalassionema* strains, with the aim to ascertain both inter-species and intra-species differences. The IR/LSC and IR/SSC boundaries were quite different among these *Thalassionema* strains (Fig. 5A). The distance between the last gene in LSC and LSC/IRb boundaries ranges from 0 to 1,020 bp, with *ycf45* located at the LSC/IRb boundaries in all *T. bacillare* cpDNAs*.* All strains’ cpDNAs had their *rps10* gene located at the IRb/LSC boundaries, and in *T. bacillare* and *T. nitzschioides* cpDNAs, another replication of *rps10* gene located at the SSC/IRa boundaries. In *T. frauenfeldii* cpDNAs, the distance between the last gene in SSC and SSC/IRa boundaries ranged from 2 to 10 bp. The distance between the first gene in LSC and IRa/LSC boundaries was 70 bp in *T. bacillare* cpDNAs and 1,646 bp in two of the *T. frauenfeldii* strains’ cpDNAs (CNS00836 and CNS00837). However, in the other *T. frauenfeldii* strain’s cpDNA (CNS00899), *ycf45* located at the IRa/LSC boundaries, that also happened in the *T. nitzschioides* cpDNA. The differences of boundaries lead to the differences of gene content in IR regions. The IR regions of *S. acus* cpDNA contained eight genes, *psbY*, *rrn5*, *rnl*, *trnA(ugc)*, *trnI(gau)*, *rns*, *ycf89* and *trnP(ugg)*. In contrast, the lengths of IR regions of *Thalassionema* cpDNAs were significantly longer and contained more genes (Fig. 5B). Seven cpDNAs all contained *rps6*, *trnC(gca)*, *psaC* and partial *rps10* in their IR regions, while three *T. frauenfeldii* cpDNAs had an extra *clpC*. Interestingly, the strain CNS00899 had longer IR regions with two extra *orf*s. Generally, the components of IR regions in cpDNA further reflected the uniqueness of the strain CNS00899.

**Fig. 6:**
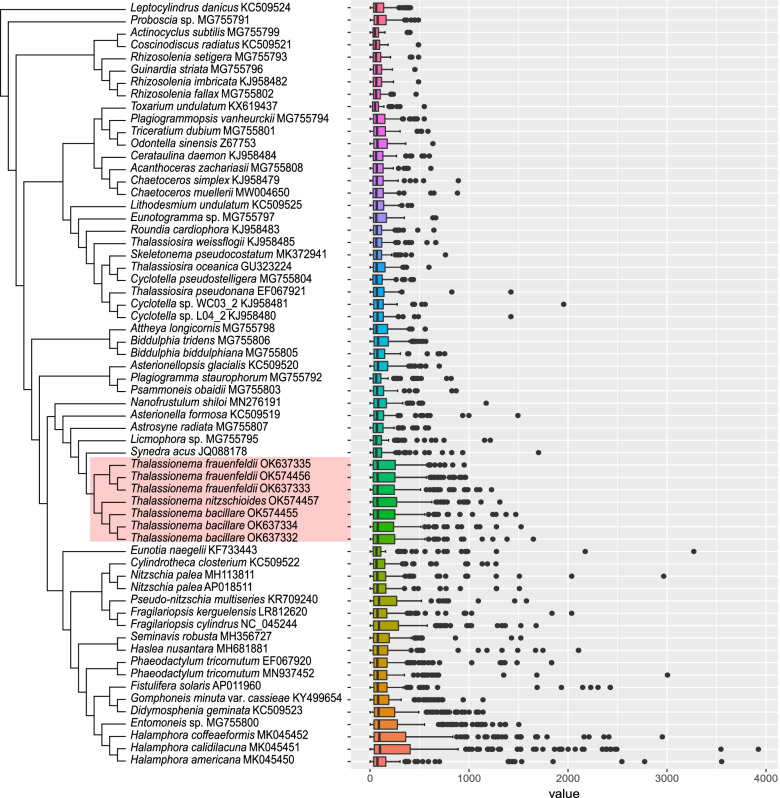
Intergenic spacers of seven *Thalassionema* cpDNAs, compared with 55 published diatom cpDNAs

Furthermore, the preservation of large intergenic spacers is also a significant feature for *Thalassionema* cpDNAs (Fig. 6). The maximum size of intergenic spacers ranged from 986 bp (in *T. bacillare* cpDNA) to 2,174 bp (in the *T. nitzschioides* cpDNA). On average, intergenic spacers in *T. frauenfeldii* cpDNAs were over 200 bp, larger than that of others (Table 1). Among the three strains of *T. frauenfeldii*, the average size of intergenic spacers was the smallest in the strain CNS00899 with the largest spacer being 1580 bp, which was much shorter than that of the other two strains (which were 2037 bp).

**Fig. 7:**
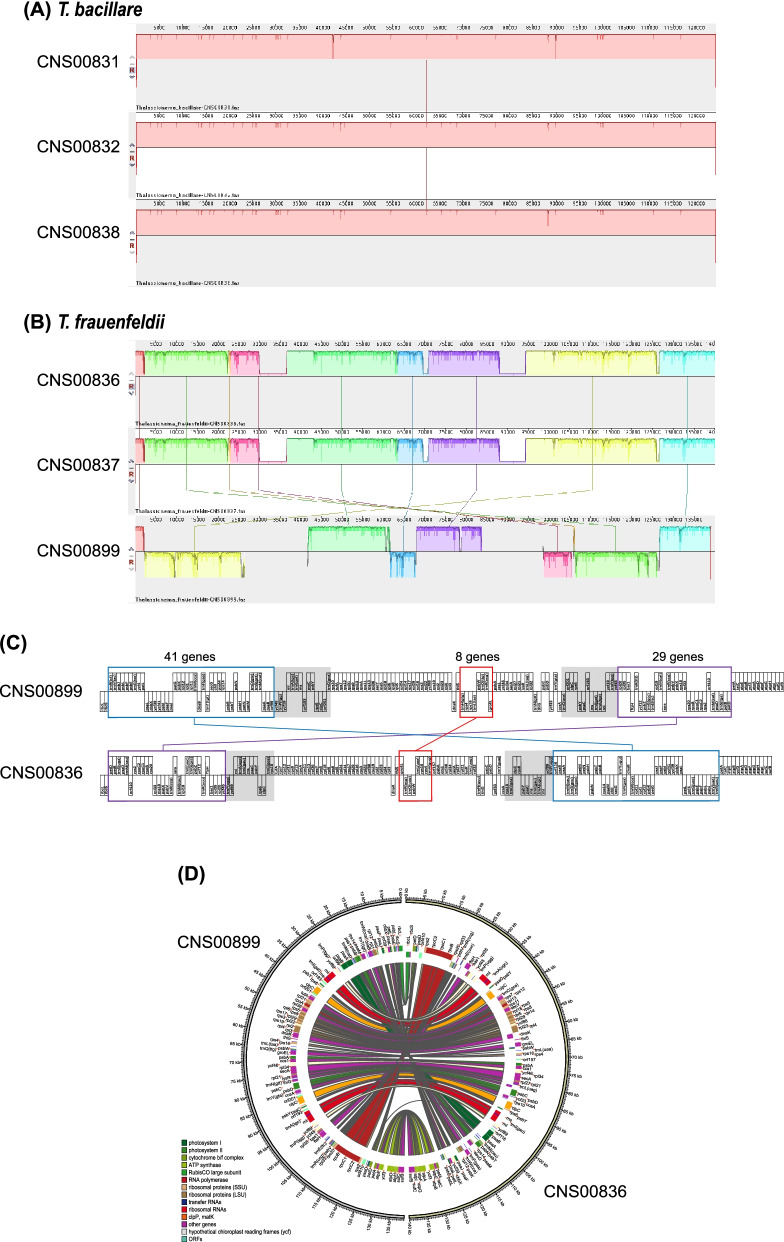
Intra-species comparative analysis of cpDNAs. (**A)** Synteny comparison of cpDNAs of three *T. bacillare* strains. (**B)** Synteny comparison of cpDNAs of three *T. frauenfeldii* strains. (**C)** Gene order comparison of two *T. frauenfeldii* (CNS00899 and CNS00836) cpDNAs. Grey boxes represent the IR regions, and same gene blocks are in the boxes of the same colors. (**D)** CIRCOS plots show synteny comparison between two *T. frauenfeldii* (CNS00899 and CNS00836) cpDNAs. Genes with the same color share similar function

While the three *T. bacillare* strains share similar cpDNA structures (Fig. 7A), the cpDNAs of *T. frauenfeldii* strains (Fig. 7B), especially between strain CNS00899 and other two *T. frauenfeldii* strains, showed substantial differences including size differences and structural differences (Fig. 7C-D). Substantial translocation and inversion events were found between CNS00899 and CNS00836 cpDNAs (Fig. 7C-D). A large translocation, along with inversion was found in two conservative gene blocks, containing 29 (enclosed in purple box) and 41 genes (enclosed in blue box) respectively, in the LSC region. Furthermore, a small inversion covering eight genes (enclosed in red box) was found in the SSC region (Fig. 7C-D). No such intra-species differences in cpDNAs has been reported previously.

### Phylogenetic analysis and divergence time estimation

**Fig. 8:**
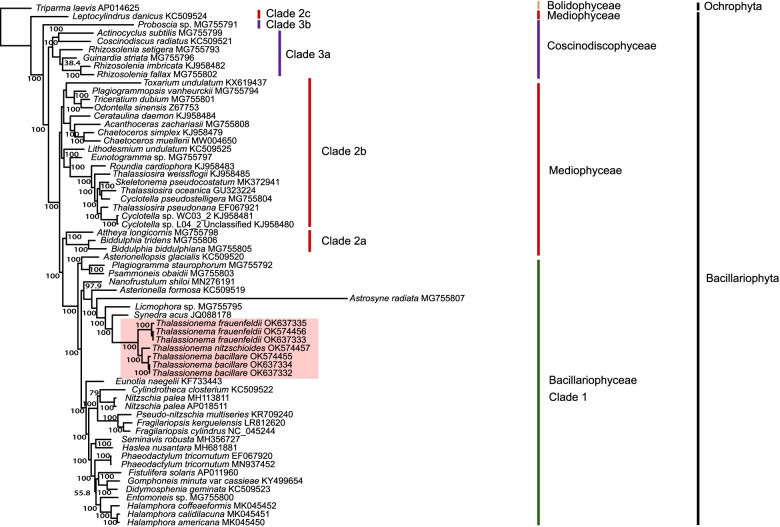
Phylogenetic tree based on maximum likelihood (ML) analysis of amino acid (aa) sequence dataset of 113 cpDNA PCGs in Bacillariophyta. The species *Triparma laevis* (AP014625) (Bolidophyceae, Ochrophyta) was used as the outgroup taxa. Numbers on the branches represent the percentage of 1000 bootstrap values

To explore phylogenetic positions of these *Thalassionema* strains in the context of Bacillariophyta, we constructed phylogenetic analysis using the amino acid (aa) sequence dataset of 113 concatenated PCGs (21,605 bp combined size) shared by cpDNAs of Bacillariophyta and Ochrophyta (Table 2). The phylogenetic tree demonstrated that Bacillariophyta species mainly formed three major clades, corresponding to the three classes including Coscinodiscophyceae, Mediophyceae and Bacillariophyceae as expected (Fig. 8). The phylogenetic relationship is consistent to previous study [18]. As expected, *Thalassionema* strains were clustered together. We also observed higher differences compared to that based on 18S rDNA, where intra-species strains could not be distinguished (Fig. 2H). In *T. frauenfeldii* species, strain CNS00836 and CNS00837 clustered more closely, while CNS00899 displayed some genetic distance. In *T. bacillare* species, the strain CNS00838 and the strain CNS00832 clustered more closely.

**Table 2:** 113 PCGs shared by cpDNAs of Bacillariophyta and Ochrophyta

Category	Genes
Photosystem I	*psaA*, *psaB*, *psaC*, *psaD*, *psaF*, *psaJ*, *psaL*
Photosystem II	*psbA*, *psbB*, *psbC*, *psbD*, *psbE*, *psbF*, *psbH*, *psbI*, *psbJ*, *psbK*, *psbL*, *psbN*, *psbT*, *psbV*, *psbW*, *psbX*, *psbY*, *psbZ*
Cytochrome b/f complex	*petA*, *petB*, *petD*, *petG*, *petL*, *petM*, *petN*
ATP synthase	*atpA*, *atpB*, *atpD*, *atpE*, *atpF*, *atpG*, *atpH*, *atpI*
RubisCO subunit	*rbcL*, *rbcS*
RNA polymerase	*rpoA*, *rpoB*, *rpoC1*, *rpoC2*
Ribosomal proteins (SSU)	*rps2*, *rps3*, *rps4*, *rps5*, *rps6*, *rps7*, *rps8*, *rps9*, *rps10*, *rps11*, *rps12*, *rps13*, *rps14*, *rps16*, *rps17*, *rps18*, *rps19*, *rps20*
Ribosomal proteins (LSU)	*rpl1*, *rpl2*, *rpl3*, *rpl4*, *rpl5*, *rpl6*, *rpl11*, *rpl12*, *rpl13*, *rpl14*, *rpl16*, *rpl18*, *rpl19*, *rpl20*, *rpl21*, *rpl23*, *rpl24*, *rpl27*, *rpl29*, *rpl31*, *rpl32*, *rpl33*, *rpl34*, *rpl35*
Other genes	*ccs1*, *ccsA*, *chlI*, *clpC*, *dnaB*, *dnaK*, *ftsH*, *groEL*, *lysR*, *secA*, *secG*, *secY*, *sufB*, *sufC*, *tatC*, *thiG*, *thiS*, *ycf3*, *ycf4*, *ycf12*, *ycf33*, *ycf39*, *ycf41*, *ycf45*, *ycf46*

**Fig. 9:**
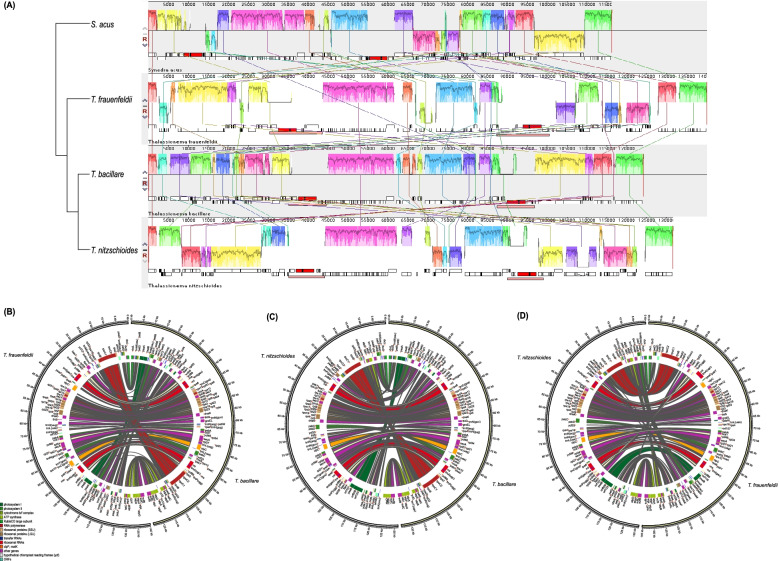
Phylogenetic analysis based on syntenic comparison of three *Thalassionema* species cpDNAs. The species *S. acus* was used as out-group taxa. (**A)** Syntenic analysis of the three *Thalassionema* species cpDNAs using Mauve. (**B-D)** Pairwise comparison of the three cpDNAs. Genes with same color share similar function

Syntenic analysis of the three *Thalassionema* species, as well as the pairwise comparison of these three species, all exhibited substantial genome rearrangement events (Fig. 9), which was different from previous studies that revealed strong collinearity among the cpDNAs of the same genus [47, 48].

**Fig. 10:**
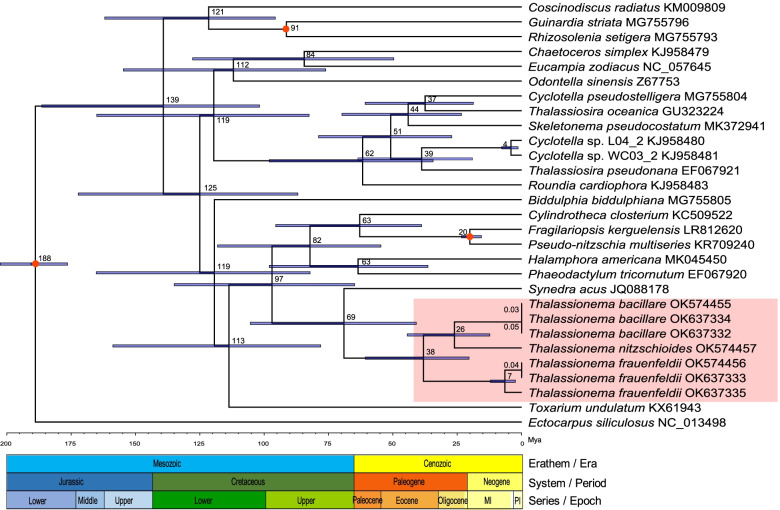
Emergence and divergence time estimation for *Thalassionema* strains. The estimation used Bayesian analysis based on the nucleotide sequences of 113 PCGs shared in 28 Bacillariophyta cpDNAs. The fossil calibration taxa are indicated with red points on the corresponding nodes. Horizontal bars represent 95% highest posterior density (HPD) values of the estimated divergence time

A total of 113 PCGs shared by 28 species were used to explore the divergence of *Thalassionema* species in the context of other diatom species. Divergence time estimation suggested that the common ancestor of the *Thalassionema* species, which formed a monophyletic clade at approximately 38 Mya, split from *S. acus* at about 69 Mya (Fig. 10). Among three *Thalassionema* species, *T. frauenfeldii* appeared at 38 Mya, while the diversification between *T. bacillare* and *T. nitzschioides* occured at 26 Mya. As expected, the strain CNS00899 split from other two *T. frauenfeldii* strains at about seven Mya (Fig. 10).

## Discussion

Diatoms are an extraordinarily diverse lineage with more than 200,000 species and cpDNA is a vital genetic material for studying their phylogenetic evolution [49, 50]. To date, there are only about 70 diatom cpDNAs being published, with many orders either underrepresented or entirely unrepresented. The small sample and incomplete varieties have impeded in-depth understanding of broad-scale patterns of evolution [17]. In this project, we constructed cpDNAs of seven *Thalassionema* strains corresponding to three common species in China for the first time. Notably, they are the first cpDNAs for any species in the order Thalassionematales that includes 35 reported species and varieties. This study not only represents an important step forward into understanding the *Thalassionema* species, but also enriches research on diatom cpDNA evolution, contributing to further exploration.

### Intra-species and inter-species variations of cpDNA sizes

Among the seven *Thalassionema* strains, three *T. bacillare* strains shared similar cpDNA size, so did the three *T. frauenfeldii* strains, which is expected [51]. The cpDNAs of different species *T. bacillare*, *T. frauenfeldii* and *T. nitzschioides* varied substantially in the length, ranging from 124,127 bp to 140,121 bp (Fig. 3, Table 1), which is also expected because the lengths of cpDNAs of different species in the same genus can be quite different, such as in genera *Thalassiosira* and *Pseudo-nitzschia* [48, 52], although cpDNAs of different species within a genus can be remarkably similar such as in genera *Skeletonema* and *Chaetoceros* [47, 53]. According to previous studies, diatom cpDNAs are particularly labile in size, with the longest cpDNA being 201,816 bp in *Plagiogramma staurophorum* (MG755792) [18], and the smallest one being only 111,539 bp in *Pseudo-nitzschia multiseries* (KR709240) [54]. Many reasons can contribute to the variations in the sizes of cpDNAs [55], and comparative analysis revealed that the variation of *Thalassionema* cpDNA lengths was driven by the combination of several reasons, including gene loss and acquisition, presence and absence of introns, IR contraction and expansion, and the variation of intergenic regions. *T. frauenfeldii* stains possess the longest cpDNAs for their longest IR regions and intergenic spacers (Table 1, Fig. 5). Although the strain *T. nitzschioides* has the shortest IR regions, the total size of cpDNA was not so small because it had an intron and relatively longer intergenic spacers (Table 1, Fig. 5). In contrast, *T. bacillare* strains have the shortest cpDNAs, not only for their shortest intergenic spacers, but also for the lack the gene *tufA* (Table 1, Fig. 4).

### Conservation of gene content, despite gene loss and transfer events

Among different strains of species *T. bacillare*, the gene contents of cpDNAs are exactly the same. Similarly, cpDNAs genes in three *T. frauenfeldii* stains are just different in several *orf*s (Table 1). The similarity was also found in different strains of the species *Phaeodactylum tricornutum* (EF067920, MN937452) and *Nitzschia palea* (MH113811, AP018511) in previous studies [56, 57]. Furthermore, gene contents of different *Thalassionema* species whose cpDNA lengths varied substantially also share high similarities. The only two differences were that *T. bacillare* cpDNAs lacked the gene *tufA* (Fig. 3, Fig. 4A) and the *T. nitzschioides* cpDNA possessed a group II intron in the gene *psaA* (Table 1). The conserved gene content in intra-genus species have been similarly discovered between *Chaetoceros muelleri* (MW004650) and *C. simplex* (KJ958479) [51], *Biddulphia biddulphiana* (MG755805) and *B. tridens* (MG755806) [18], *Thalassiosira weissflogii* (KJ958485) and *T. pseudonana* (EF067921) [51, 57]. In some genera, however, cpDNAs genes can be quite different in different species, such as the genus *Fragilariopsis* (LR812620, NC_045244) [58] and *Rhizosolenia* (KJ958482, MG755802, MG755793) [18, 51]. These differences may reflect species-specific gene loss, which may reflect differences in species divergence.

Compared to the close relative *S. acus*, the cpDNAs of three *Thalassionema* species all lacked the genes *petF*, *psaE*, *psaI*, *syfB*, *ycf35*, and *ycf66* (Fig. 4A), and more *Thalassionema* species should be studied in the future to estimate whether these events occurred in their common ancestors. Among these genes, *petF*, *psaE*, and *psaI* were found transferred to nuclear genomes, while *syfB*, *ycf35*, and *ycf66* were proven to be lost. In addition, cpDNAs of *Thalassionema* species and *S. acus* lacked genes including *acpP*, *ilvB*, *ilvH*, *chlB*, *chlL*, *chlN*, *petJ*, *ycf90* and *ycf91*, all of which were found missing from cpDNAs of some species previously [17, 18, 51, 57]. None of these genes was found in nuclear genomes of all seven *Thalassionema* strains. Indeed, massive numbers of gene losses or transfers have been identified in diatom cpDNAs, reflecting a dynamic history across a broad range of phylogenetic depths, suggesting as a pervasive source of genetic change that potentially causes adaptive phenotype diversity [17, 59].

### Substantial genome rearrangement events in *Thalassionema* species

Diatom cpDNAs appear to be highly rearranged, even between close relatives [57, 60]. Although in some diatom genera cpDNAs of different species revealed strong collinearity [47, 48], we discovered substantial genome rearrangement events in cpDNAs of all three *Thalassionema* species constructed in this project (Fig. 9). Notably, rearrangements were found to be restricted to either the LSC or the IR-SSC-IR regions without involving gene exchange between regions, consistent to previous studies [60].

What was surprising was the observation that cpDNAs of different strains of the same species *T. frauenfeldii* showed substantial genome rearrangement events, including translocation and inversion events between CNS00899 and CNS00836 cpDNAs (Fig. 7C-D). In addition to their different structures, the cpDNAs of these two strains also showed differences in cpDNA sizes and sizes of IR regions and intergenic spacers. This is the first case showing substantial structural differences in cpDNAs among strains of a same species. Previous studies have found that the species *T. nitzschioides* was highly variable with eight variants [13, 14], suggesting that large genomic differences may exist among different strains of a same *Thalassionema* species such as we have observed for *T. frauenfeldii*. Alternatively, the species *T. frauenfeldii* may actually represent multiple cryptic species as observed for *Alexandrium tamarense*, which was split into five species that showed genetic differences [61].

It has been suggested that gene order can be used in wide-range phylogenetic studies [62]. However, the pathways of gene rearrangement are so complex that only more extensive sampling of cpDNAs would make rigorous analysis possible [57], suggesting that more *Thalassionema* cpDNAs are needed to gain further insight into the genome rearrangements.

### Phylogenetic position and speciation of *Thalassionema* species

Phylogenetic analysis based on core genes of cpDNAs were consistent to previous studies, and supported the current taxonomic status of *Thalassionema* species [18]. According the divergence time estimation, we found the emergence of diatoms occurred in 188 Mya, similar to previous reports [63]. The split of *Thalassionema* species from *S. acus* occurred at about 69 Mya and the divergence of *Thalassionema* species, which formed a monophyletic clade, occurred at approximately 38 Mya (Fig. 10), consistent to previous report [7].

## Supplementary Information

**Additional file 1.** **Table S1**.  Amount of clean reads of seven samples used for analysis.

## Data Availability

The chloroplast genomes sequences of seven strains (CNS00831, CNS00832, CNS00836, CNS00837, CNS00838, CNS00894, CNS00899) have been deposited in GenBank under accession numbers OK574455, OK637332, OK574456, OK637333, OK637334, OK574457 and OK637335, respectively.

## Abbreviations

cpDNA: Chloroplast genome
HABs: Harmful algal blooms
PCGs: Protein-coding genes
IRs: Inverted repeat regions
ML: Maximum likelihood
LSC: Large single copy
SSC: Small single copy
ORF: Open reading frame
EGT: Endosymbiotic gene transfer
Aa: Amino acid
HPD: Highest posterior density
