# Supplementary material for: Comparative analysis of Thalassionema chloroplast genomes revealed hidden biodiversity
Source: BMC Genomics. 2022 Apr 27;23:327. doi: 10.1186/s12864-022-08532-6 (PMC9044688; doi:10.1186/s12864-022-08532-6)
Supplement: Supplementary file 1 — Additional file 1. Table S1. Amount of clean reads of seven samples used for analysis. [file 12864_2022_8532_MOESM1_ESM.docx]

Table S1

Amount of clean reads of seven samples used for analysis

| **Strain** | **Reads number (single)** | **Estimated genome size /bp** | **Coverage** |
| --- | --- | --- | --- |
| CNS00831 | 40,387,840 | 274,420,806 | 44x |
| CNS00832 | 37,788,042 | 264,678,469 | 43x |
| CNS00836 | 34,551,691 | 145,248,316 | 71x |
| CNS00837 | 34,691,808 | 106,134,920 | 98x |
| CNS00838 | 35,086,777 | 263,033,829 | 40x |
| CNS00894 | 35,860,303 | 457,853,896 | 23x |
| CNS00899 | 36,146,315 | 238,998,366 | 45x |
